# Smooth Muscle Strips for Intestinal Tissue Engineering

**DOI:** 10.1371/journal.pone.0114850

**Published:** 2014-12-08

**Authors:** Christopher M. Walthers, Min Lee, Benjamin M. Wu, James C. Y. Dunn

**Affiliations:** 1 Department of Bioengineering, University of California Los Angeles, Los Angeles, California, United States of America; 2 Division of Advanced Prosthodontics, Biomaterials, and Hospital Dentistry, University of California Los Angeles, Los Angeles, California, United States of America; 3 The Weintraub Center for Reconstructive Biotechnology, University of California Los Angeles, Los Angeles, California, United States of America; 4 Department of Surgery, University of California Los Angeles, Los Angeles, California, United States of America; Temple University School of Medicine, United States of America

## Abstract

Functionally contracting smooth muscle is an essential part of the engineered intestine that has not been replicated *in vitro*. The purpose of this study is to produce contracting smooth muscle in culture by maintaining the native smooth muscle organization. We employed intact smooth muscle strips and compared them to dissociated smooth muscle cells in culture for 14 days. Cells isolated by enzymatic digestion quickly lost maturity markers for smooth muscle cells and contained few enteric neural and glial cells. Cultured smooth muscle strips exhibited periodic contraction and maintained neural and glial markers. Smooth muscle strips cultured for 14 days also exhibited regular fluctuation of intracellular calcium, whereas cultured smooth muscle cells did not. After implantation in omentum for 14 days on polycaprolactone scaffolds, smooth muscle strip constructs expressed high levels of smooth muscle maturity markers as well as enteric neural and glial cells. Intact smooth muscle strips may be a useful component for engineered intestinal smooth muscle.

## Introduction

Short bowel syndrome (SBS) is a significant clinical complication that occurs when there is insufficient length of functional intestine, leading to malabsorption [1]. Standard treatments for SBS include total parenteral nutrition, intestinal transplantation, and intestinal lengthening; however severe complications and reduced quality of life limit their effectiveness [1]–[3]. Intestinal tissue engineering is an innovative approach for the treatment of SBS.

The gastrointestinal tract is responsible for digestion, nutrient and water absorption, and excretion of waste [4]. Within the digestive tract are several specialized tissues, including the smooth muscle, that are responsible for mixing luminal contents for absorption and propelling contents toward the rectum. Between the layers of smooth muscle is the myenteric plexus that coordinates smooth muscle contraction and intestinal motility [4], [5]. Pacemaker activity originates in the interstitial cells of Cajal (ICCs) and the pathway of conduction to intestinal smooth muscle is controversial [6]. Smooth muscle contraction is integrated by signals from many sources and may conduct through the enteric nervous system, through ICCs, or in another way entirely [7]–[9]. Glial cells form networks with smooth muscle cells [10], and the loss of glia decreases smooth muscle motor function [11]

Engineered intestine requires the expansion and incorporation of several cell types into structured layers. The smooth muscle layers are particularly challenging to replicate. Isolated smooth muscle cells (ISMC) lose maturity, particularly the expression of smooth muscle myosin heavy chain, even after brief culturing [12]–, leading to a loss of contractile strength [16] and loss of excitation/contraction coupling [17]. This altered state of ISMC maturity is known as the “synthetic phenotype” and is marked by increased proliferation coupled with decreased differentiation [14]. While cell growth is desirable in tissue engineering, the main disadvantage of dissociated ISMC is the loss of an appropriate cell niche [18].

Attempts to develop tissue-engineered smooth muscle for the gastrointestinal tract have been unable to reproduce the contraction strength associated with healthy tissue [19], [20]. This is true for other tissue-engineered muscles including skeletal muscle [21], cardiac muscle [22], vascular smooth muscle [23], sphincteric smooth muscle [24], and bladder smooth muscle [25]. Many methods to increase cultured smooth muscle maturity have been studied including the use of growth factors [15], [24], [26], scaffolds [27], [28], stem cells [25], co-culture [29], bioactive materials [30], and physiological stimulation [31], [32], but the contraction remains poor. Maintaining the close association of smooth muscle cells with neuroglial cells and ICCs may be necessary for functioning smooth muscle cells.

The objective of this study was to engineer intestine smooth muscle from intact strips of smooth muscle and compare them to dissociated ISMC. We evaluated the expression of smooth muscle maturity markers, enteric nervous markers, and periodic contraction of cultured cells and tissues. Finally, we evaluated the behavior of ISMCs and muscle strips after implantation on polymeric scaffolds.

## Materials and Methods

### A) Muscle strip (MS) and ISMC procurement

Pregnant Lewis rats were purchased from Charles River Laboratories (Wilmington, MA) to give birth to wild-type pups. Transgenic Lewis rat pups that expressed green fluorescent protein (GFP) were bred in a colony managed by the Department of Laboratory Animal Medicine and approved by the UCLA Institutional Review Board and Office of Animal Research Oversight protocol #2004-183. The UCLA facility is an AALAC-accredited facility and experiments strictly followed the recommendations within the Guide for the Care and use of Laboratory Animals supplied by the National Institutes of Health. All efforts were made to minimize animal suffering. The isolation of MS from intestine was described previously (12). Briefly, pups were sacrificed at day 3–5 postpartum using an isofluorane overdose and confirmed by decapitation. The removed intestines were kept on ice in Hank's Buffered Saline Solution lacking magnesium and calcium (HBSS, Life Technologies, Carlsbad, CA) with 1x antibiotic-antimycotic (ABAM, Life Technologies). The circular and longitudinal layers of smooth muscle were gently separated together in one strip from the intestine using forceps. Half of the MS were minced using a razor blade until they were less than 500 µm in size. The other half of the MS were used to obtain dissociated ISMC as described previously (12) employing a 1 mg/ml solution of collagenase IV (Sigma, St. Louis, MO) in HBSS for 30 minutes at 37°C. Cells were suspended in DMEM containing 10% fetal bovine serum and 1x ABAM and were filtered through a 70 micron nylon filter (Corning, Corning, NY) prior to culture.

### B) Cell culture conditions

24-well culture plates (Corning) were coated overnight prior to cell seeding at 37°C with 50 µg/ml of type I bovine collagen (Purecol, Advanced Biomatrix, San Diego, CA) diluted in phosphate buffered saline (PBS, Life Technologies). Wells were rinsed twice in PBS prior to seeding. ISMC were counted with a hemacytometer and were seeded at 50,000 cells/cm^2^. MS were plated at a density of 20 minced pieces per well in 500 µl of culture medium. Both ISMC and MS were kept in a 37°C humidified incubator with 10% carbon dioxide. The culture medium was changed every other day after rinsing with PBS.

### C) Immunofluorescence

S100 protein is expressed by glial cells in the intestine [10] and is used as a glial cell marker. The enteric neural cells express β (III)-tubulin (BTUB) (30). Cultured ISMC and MS were fixed in formalin for 1 hour at room temperature. Samples were incubated with the following primary antibodies: monoclonal mouse anti-α smooth muscle actin (SMA, 1∶50 dilution, Dako, Carpinteria, CA), monoclonal mouse anti-desmin (DES, 1∶50, Dako), monoclonal mouse anti-smooth muscle myosin heavy chain (MHC, 1∶50, Santa Cruz Biotechnology, Dallas, TX), polyclonal rabbit anti-S100 (1∶200, Dako), and monoclonal mouse anti-β (III) tubulin (Abcam, 1∶200, Cambridge, England). After 16 hours of incubation, samples were incubated in a 1∶200 dilution of Alexafluor 488 (green) goat anti-mouse or Alexafluor 594 (red) anti-rabbit IgG (Life Technologies). VectaMount containing DAPI (Vector) was used to visualize the nuclei. All images and videos were taken with an Olympus IX71 microscope with CellSens software (Olympus, Center Valley, PA).

### D) mRNA expression

mRNA was isolated from lysed ISMC and MS using the RNeasy kit (Qiagen, Germantown, MD) and Qiashredder kit (Qiagen) following the manufacturer's protocol. Real-time PCR was performed using a PCR master mix and Quantitect Probe RT-PCR kit (Qiagen), and the ABI PRISM 7700 Sequence Detection System (Applied Biosystems, Invitrogen). Primer/probe mixtures for SMA, DES, MHC, S100A, BTUB, and glyceraldehyde 3-phosphate dehydrogenase (GAPDH) were purchased from Applied Biosystems. Samples were normalized to GAPDH expression. Fresh MS from pups served as the reference for comparison of mRNA expression level.

### Measurement of depolarization and contraction of cultured smooth muscle

After culturing ISMC and MS for 14 days, wells were rinsed once with PBS and were incubated in 400 µl of a stock solution composed of 1 part Fluo-4 Direct Calcium Assay (Life Technologies) and 1 part cell-culture medium. Fluo-4 Direct emits green fluorescent light in the presence of intracellular calcium. During depolarization, calcium increases intraceullarly in smooth muscle cells and fluorescent intensity of the calcium indicator increases measurably from baseline levels. After cells repolarize, the fluorescence is reduced again to baseline – this cycle is repeated without loss of fluorescent intensity for several hours until exhaustion of the calcium indicator and without need to replace the media/calcium indicator stock solution. Wells containing ISMCs or MS were incubated for 60 minutes at 37°C and then allowed to return to room temperature for an additional 30 minutes while wrapped in aluminum foil to prevent photo-bleaching. Fluorescent images were taken at an excitation wavelength of 494 nm and an emission wavelength of 516 nm.

Fluorescent videos were analyzed using a custom MATLAB program. Each video was condensed by capturing only every 4^th^ frame. A region of interest was highlighted and the average fluorescent intensity within the highlighted region was calculated for each frame to observe calcium concentration. The intensity values from the first image in each stack were then subtracted as baseline intensity from each subsequent frame to create a normalized intensity profile compared to the initial image. Changes in intensity are rendered in the MATLAB colormap “gray”, with increases in calcium concentration shown in grayscale as an increase (black), decrease (white), or no change (gray) in intensity from the initial image.

A fast Fourier transform (FFT) was performed on the mean intensity of the region selected by the user, and the largest non-zero peak of the power within the frequency and period domain was selected.

### E) Response to pharmacological stimulation

The cholinergic agonist carbachol and antagonist atropine were administered to test pharmacological responses to cholinergic stimulation after 14 days in culture. MS and ISMCs were incubated for 1 hour in 500 µl of Fluo-4 Direct alone or Fluo-4 Direct with 150 µM atropine (Thermo Fisher Scientific) as described above. After samples were recorded in the presence of calcium indicator with the atropine still added as a baseline recording of calcium intensity, a 250 µl of a 3 µM solution of cholinergic agonist carbachol (Thermo Fisher Scientific) in PBS was added to the samples and recorded to observe changes in calcium indicator intensity within the cultures.

### F) Scaffold fabrication and seeding

Fabrication and collagen coating of poly ε-caprolactone (PCL) scaffolds were described previously (31). After forming the electrospun mat, a pattern of 250-µm pores drawn with Adobe Illustrator was cut from electrospun mat using a VersaLASER 2.3 laser cutting system (Universal Laser Systems, Scottsdale, AZ) in to a 25 mm by 9.5 mm rectangular scaffold.

Primary GFP-expressing ISMC were seeded at 250,000 cells/cm^2^ on collagen-coated PCL scaffolds fitted in to a custom designed polydimethylsiloxane (PDMS, Dow Corning, Midland, MI) seeding tray as determined by earlier studies [33]. Primary GFP-expressing MS were seeded at a density of approximately 50 minced pieces in 500 µl per scaffold. Every two days, culture medium was changed after rinsing with PBS. Scaffolds were maintained for two weeks in a 37°C humidified incubator with 10% carbon dioxide before implantation.

### G) Surgical methods and implantation

On the day of implantation, ISMC- or MS-seeded scaffolds were rinsed with PBS and were wrapped twice around a sterile #6 silicone catheter (Bard, Covington, GA). The scaffold was sutured to the catheter to prevent unwrapping, and ends were left open to allow maximum vascular infiltration. After wrapping, scaffolds were stored on ice in culture medium until implantation. All surgical procedures were performed in accordance with UCLA's Animal Research Committee protocol # 2004-197. The surgical procedure was described in detail in a previous publication [34]. Rats were anesthetized in accordance with protocols described by UCLA's Department of Laboratory Animal Medicine.

### H) Implant immunohistochemistry

Implants were retrieved after 14 days and each scaffold was cut in to four equal-sized cylinders and fixed in formalin for histology. Explanted scaffolds were fixed in formalin overnight, embedded in paraffin, and sectioned by UCLA's Translational Pathology Core Laboratory. The immunohistochemistry procedure has been reported previously [35]. Sectioned samples were incubated overnight in primary antibody as described above. Additionally, GFP-expressing cells were incubated with monoclonal mouse anti-green fluorescent protein (GFP, 1∶50, Clontech, Mountain View, CA). Slides were coverslipped with VectaMount containing DAPI.

### I) Statistics

Differences between groups were evaluated using the Student's *t*-test. A *p*-value <0.05 was considered statistically significant.

## Results

### A) Smooth muscle culture

**Figure 1:**
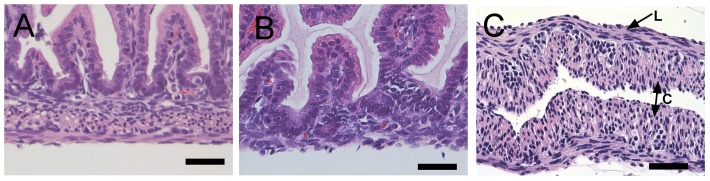
Hematoxylin and eosin histology of neo-natal intestine. At 3–5 days post-partem, normal rat intestine (A) was harvested and stripped of muscle (B). The resulting muscle strip (C) was cultured along with ISMCs isolated from the muscle strip enzymatically. Both circular (C) and longitudinal (L) smooth muscle was visible in the whole intestine and isolated MS. 400x magnification, 200-µm scale bar.

Smooth muscle layers were separated from intact mucosa and submucosa by physical stripping of the intestine (Fig. 1). Both circular and longitudinal layers of the smooth muscle were separated in to strips of muscle, which were used for ISMC isolation or MS culture (Fig. 1C).

**Figure 2:**
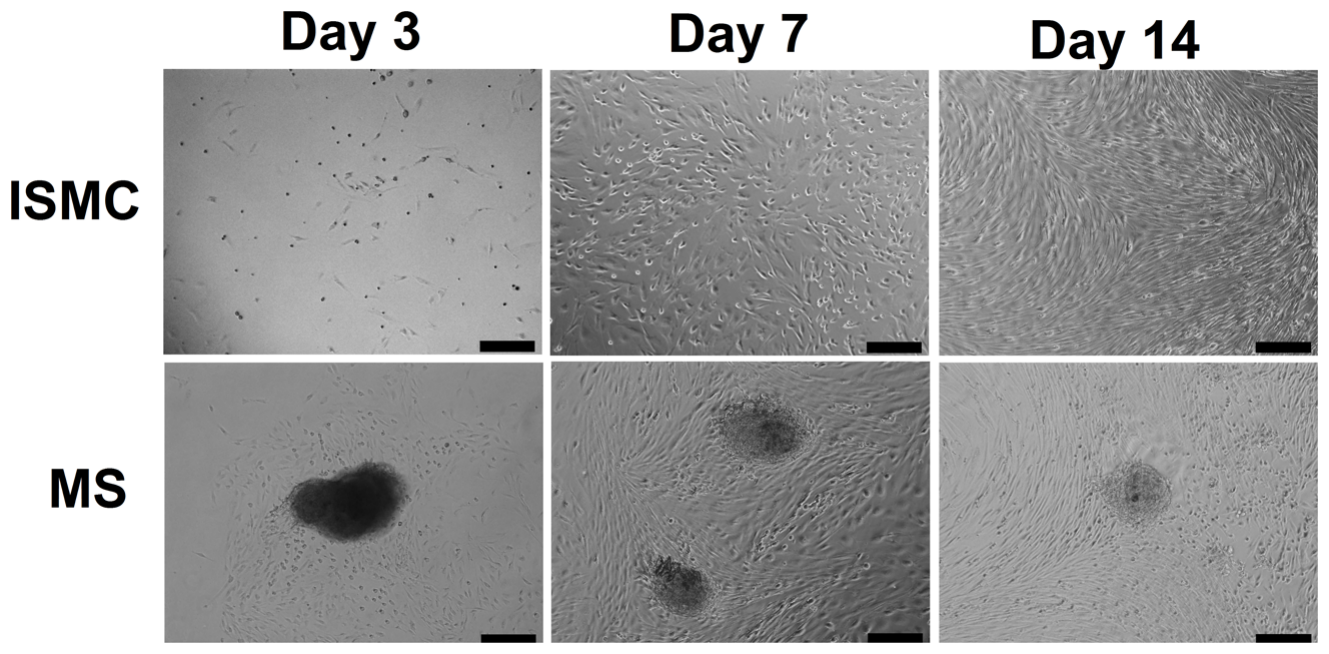
Phase contrast images of ISMCs and MS grown in culture for 3, 7, and 14 days after harvest from neonates. Smooth muscle cells (top row) were evenly dispersed after seeding and reached confluency at about 14 days in culture. Adherent MS (bottom row) were about 200-µm in diameter with cells emanating outward on the culture surface until reaching confluency after approximately 14 days in culture. 100x magnification, 200-µm scale bar.

Cultures of ISMC and MS were imaged with phase contrast microscopy to visualize cell morphology (Fig. 2). Cultures of MS formed a network of cells emanating from each piece of smooth muscle. Cells from cultured MS proliferated and migrated outwards until they merged with cells from a nearby MS. In contrast, ISMC from dissociated MS were initially more evenly distributed across the culture and had a spindle-shaped morphology. As cells grew to near confluence between 7 and 14 days, ISMC formed a hill-and-valley pattern.

### B) Immunofluorescence of cultured cells

**Figure 3:**
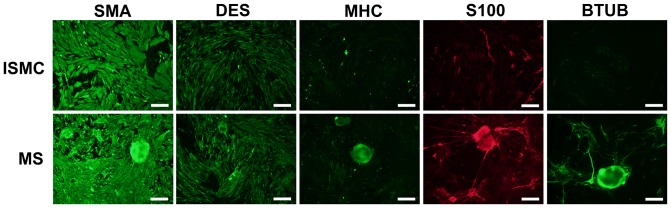
Immunofluorescence of cultured cells after 14 days. Smooth muscle cells and muscle strips were grown in culture for 14 days and stained for markers of smooth muscle, glial, and neural lineage. SMA and DES immunofluorescence confirmed smooth muscle lineage in both cultures. Both samples lacked MHC, a marker of smooth muscle maturity. Cultured MS but not cultured ISMCs contained S100 and BTUB, markers for enteric glial and neural cells. 100x magnification, 200-µm scale bar.

**Figure 4:**
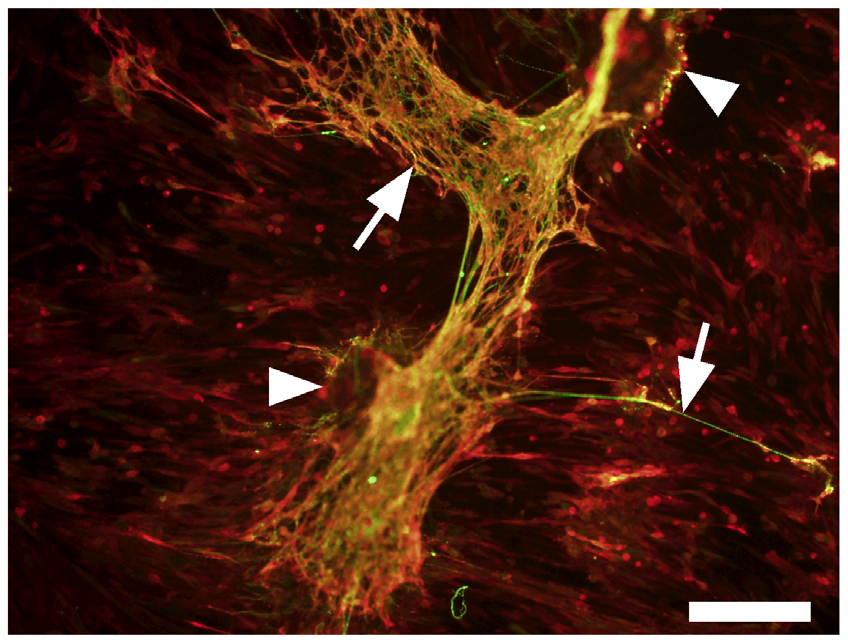
Co-immunofluorescence of S100 and BTUB in MS at Day 14. Overlapping networks of S100 (red) and BTUB (green) in cultured MS was observed. Arrows point to BTUB-positive extensions from the MS, arrowheads point to MS. 100x magnification, 200-µm scale bar.

Cultured ISMC and MS were immunofluorescently labeled to visualize expression of markers for smooth muscle and enteric neural cells (Fig. 3). By day 14, the majority of cultured ISMC and cells that grew from MS expressed SMA, and approximately half of the cells in both groups expressed DES. Less than 10% of the cells expressed MHC, suggesting de-differentiation of cultured ISMC. Approximately 10% of cultured ISMC and cells from the MS expressed S100. Strikingly, whereas 10% of cells from cultured MS expressed BTUB, cultured ISMC did not express BTUB. In MS cultures, cells that expressed S100 were often adjacent cells that expressed BTUB, suggesting that glial and neuronal cells were closely associated with each other (Fig. 4). Long, thin processes from BTUB-expressing cells extended to clustered networks of other BTUB and S100 expressing cells.

### C) Expression of mRNA in cultured ISMC and MS

**Figure 5:**
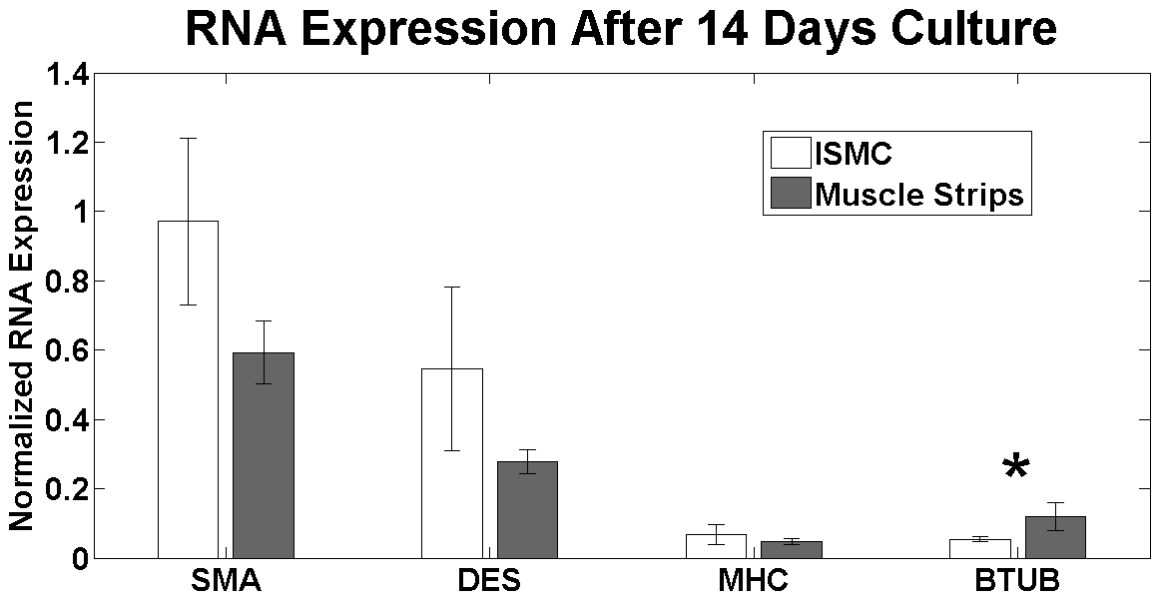
mRNA expression of cultured ISMC and MS 14 for after neonatal harvest. There was no significant difference in mRNA expression for smooth muscle markers between cultured ISMC and MS, including SMA, DES, and MHC. There was a significant increase in mRNA expression of BTUB in cultured MS (p<0.05, *; n = 6). Samples were normalized to smooth muscle from the harvest with GAPDH as a control gene.

ISMC and MS culture was examined for expression of smooth muscle, glial, and neural mRNA compared to mRNA expression in MS from the day of initial neonatal harvest (Fig. 5). No significant differences existed in ISMC and MS mRNA levels for SMA, DES, MHC, S100, or BTUB initially (data not shown). Differences in mRNA expression at day 14 between ISMC and MS cultures were congruent to the immunostaining results. The levels of mRNA for DES and MHC decreased by day 14 in both cultured ISMC and MS groups but there was not a significant difference between these markers in day 14 ISMC or MS culture. Expression of BTUB mRNA was significantly greater in cultured MS compared to cultured ISMC (p<0.05, n = 6). After culturing for 14 days, S100 mRNA expression was increased to 2.1±0.4 and 1.9±0.5 for ISMC and MS cultures, respectively, but there was no difference between groups.

### D) Contractile assessment

**Figure 6:**
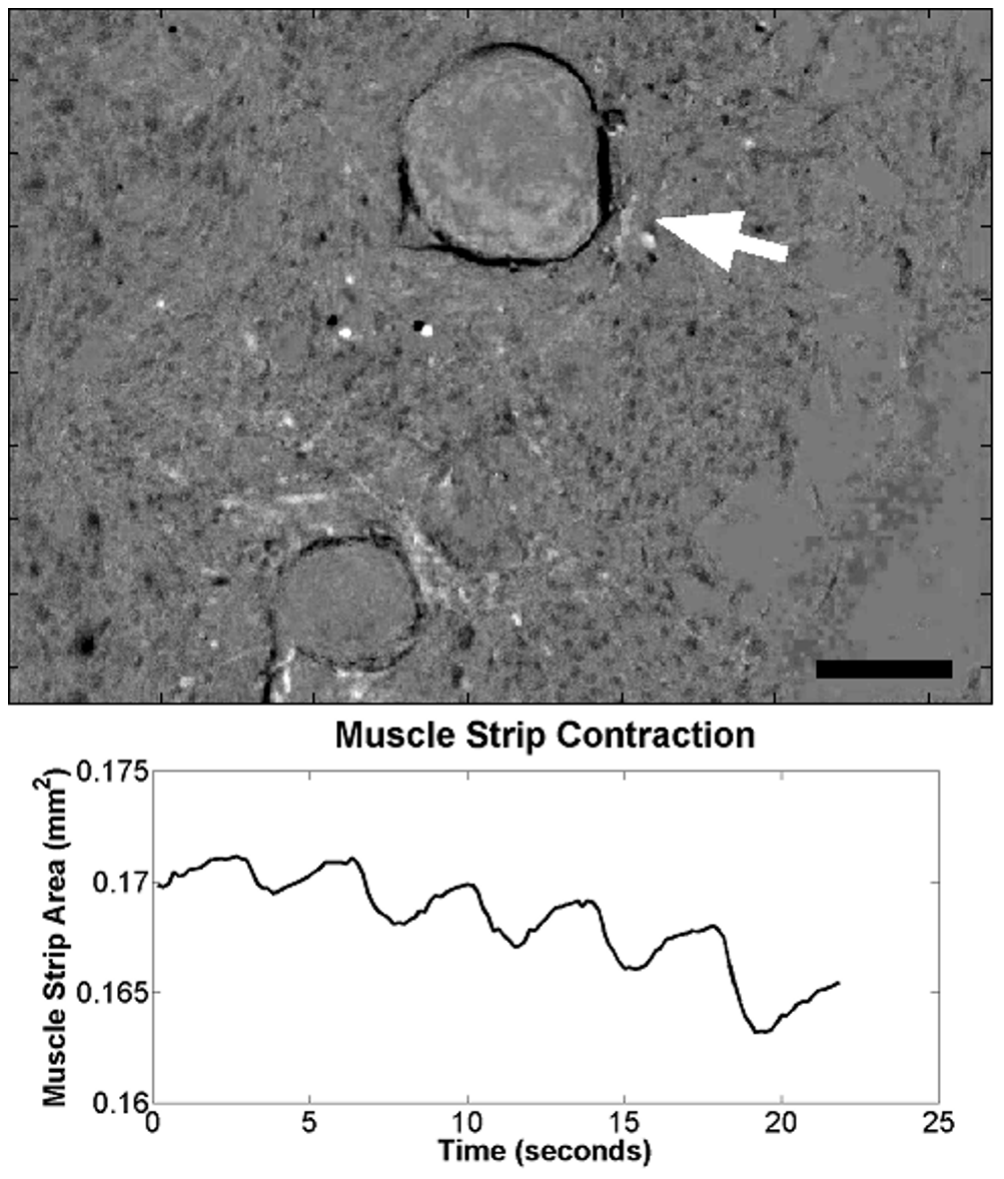
Contraction of cultured MS 14 days after neonatal harvest. A roughly 10% change in projected area was observed on a MS cultured for 14 days, showing the retained contractile function of cultured MS (S1 Video). White arrow points to observed MS, 100x magnification, 200-µm scale bar.

**Figure 7:**
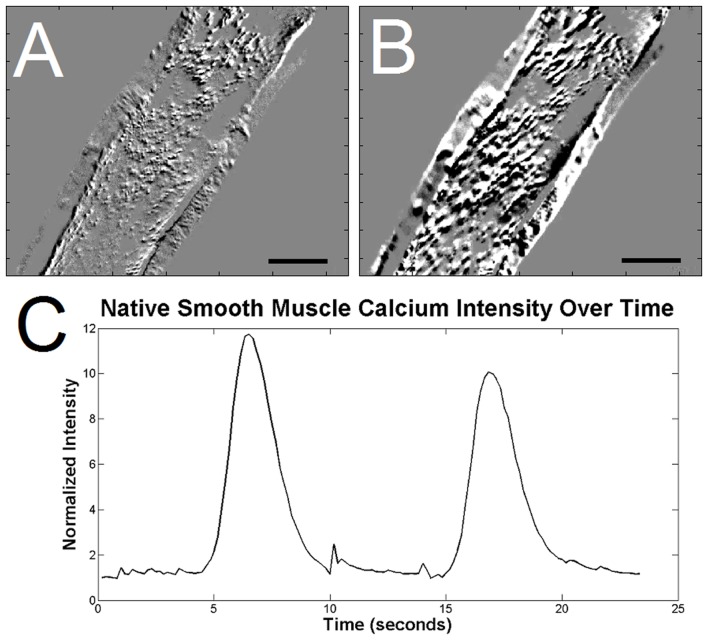
Calcium indicator intensity in native smooth muscle stripped from day 5 rat pup. An increase in calcium indicator intensity is in native smooth muscle just prior to depolarization (A) and 0.7 seconds later (B), after depolarization. Regions of black indicate depolarization, while regions of white are decreases in calcium intensity as an artifact of the smooth muscle movement during contraction. 100x magnification, 200-µm scale bar. A plot of calcium indicator intensity (C) within the native smooth muscle show the intense, periodic waves of depolarization.

**Figure 8:**
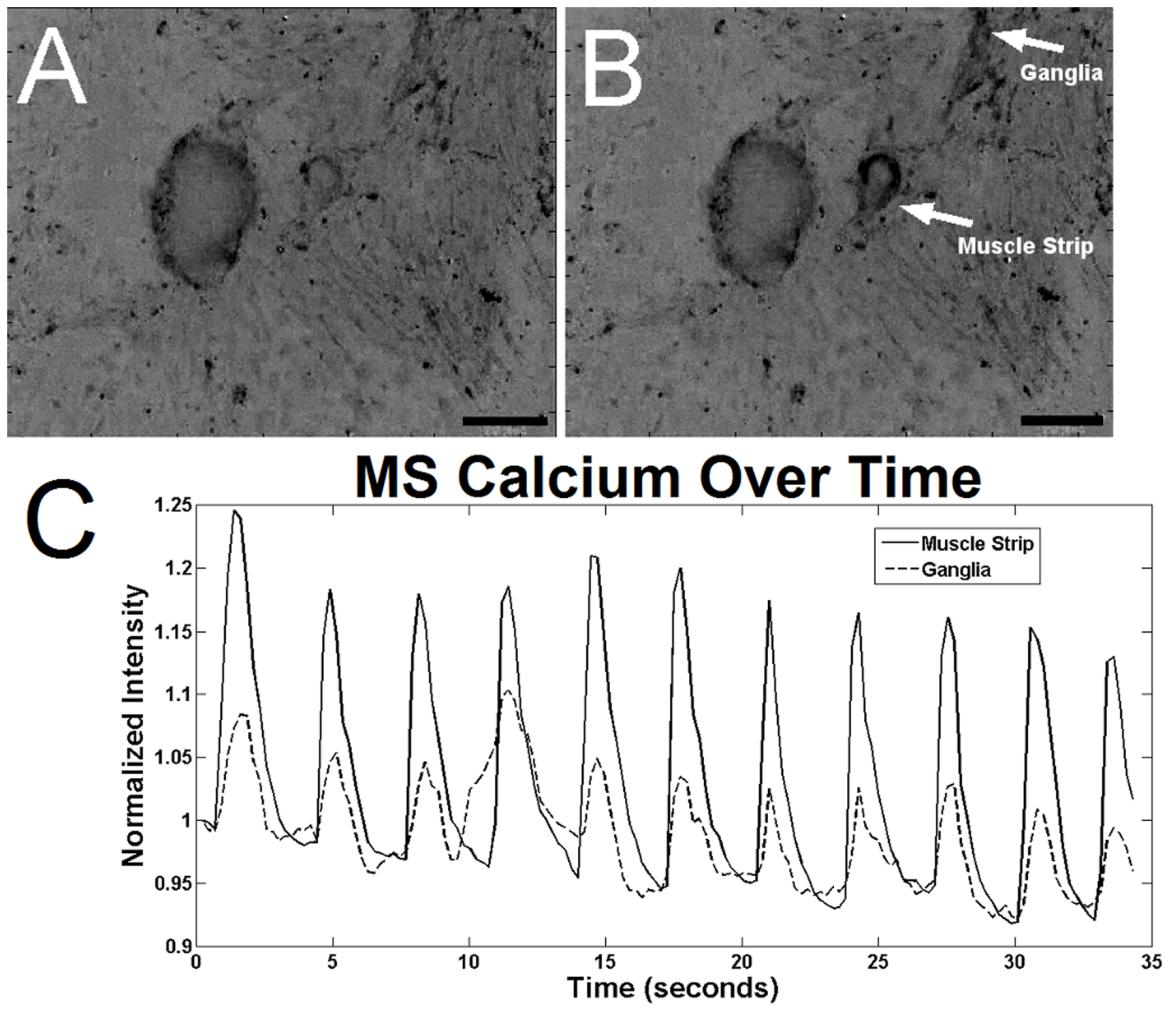
Calcium indicator intensity in cultured MS after 14 days. An increase in calcium indicator intensity is observed between images of the MS just prior to depolarization (A) and 0.7 seconds later (B), after depolarization (S2 Video). Muscle strips and the surrounding cells that formed a “ganglia” are labeled. Regions of black indicate depolarization. 100x magnification, 200-µm scale bar. A plot of calcium indicator intensity (C) within MS and the “ganglia” show the periodic depolarization waves in the MS culture.

**Figure 9:**
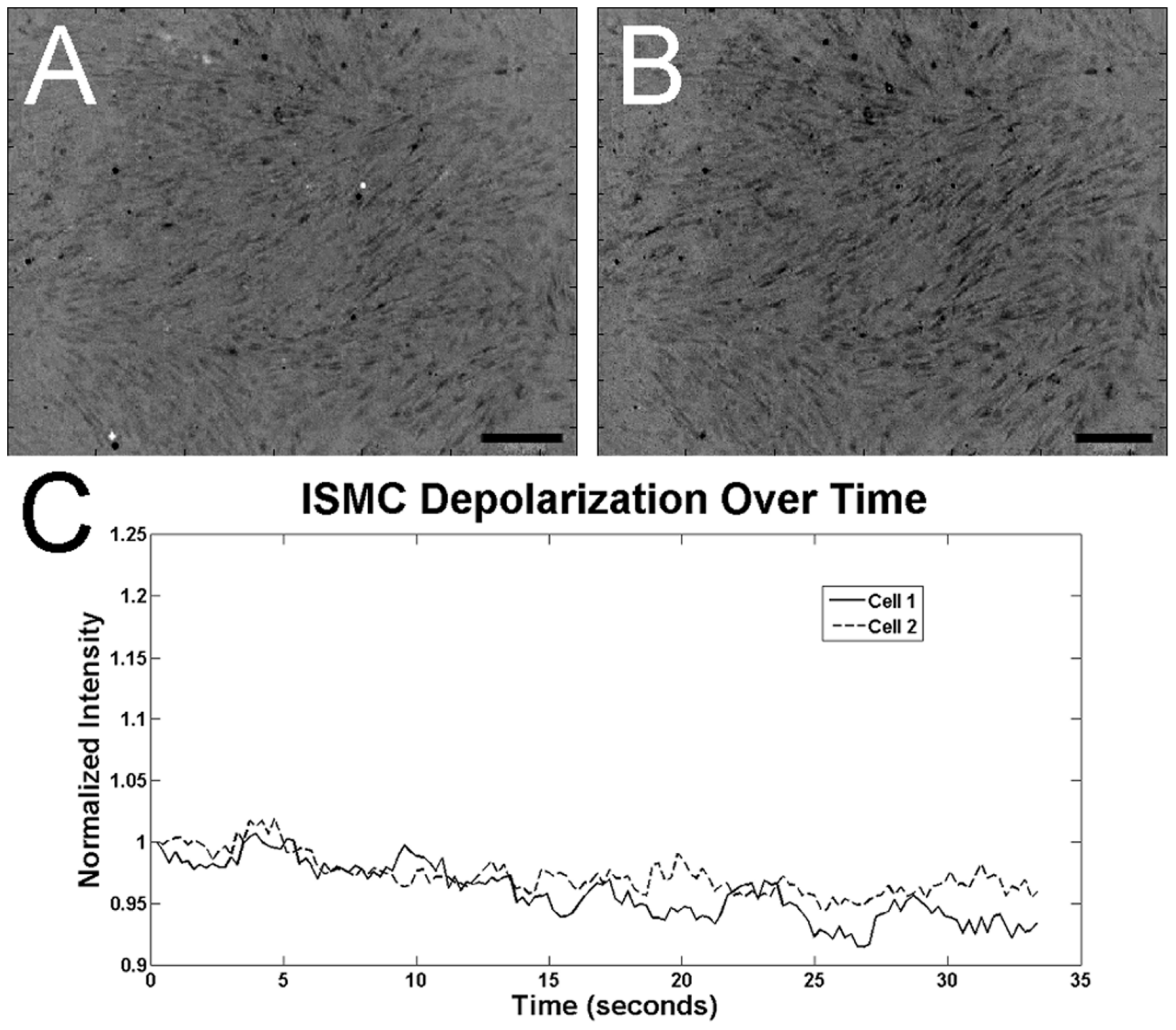
Calcium indicator intensity in cultured ISMC after 14 days. The changes in calcium indicator of ISMC were less intense than in MS in an image just prior to (A) and 0.7 seconds after depolarization (B). Calcium intensity fluctuations (C) were not periodic (S3 Video). 100x magnification, 200-µm scale bar.

To determine the contractile function of cultured ISMC and MS, cells were loaded with a fluorescent intracellular calcium indicator. MS visibly contracted in a periodic manner by changing over 10% of their projected area, suggesting the presence of mature smooth muscle cells within the cultured MS (Fig. 6; S1 Video). This periodic contraction was qualitatively less than the maximum contraction observed with pharmacological stimulants such as potassium chloride or carbachol, but similar to the ∼30% change amplitude of contraction in acetylcholine-induced fresh smooth muscle [36]. Native stripped intestinal smooth muscle contracted visibly and with a large flux of calcium intensity (Fig. 7). Cells in the MS group also exhibited periodic fluctuation of intracellular calcium level (Fig. 8; S2 Video) that closely approximated the contractile periodicity of native intestine. At day 7, the calcium oscillation had an average period of 3.1±1.0 seconds in the cultured MS group (n = 5). By day 14, the period was 2.9±0.3 seconds for the MS group (n = 5). The consistent, periodic calcium fluctuations suggested a functioning pacemaker system within the cultured MS. Furthermore, calcium waves migrated across the MS cultures, possibly via conduction through functional neuronal connections. In contrast, cultured ISMC grown in the same conditions did not show periodic changes in intracellular calcium level (Fig. 9; S3 Video).

**Figure 10:**
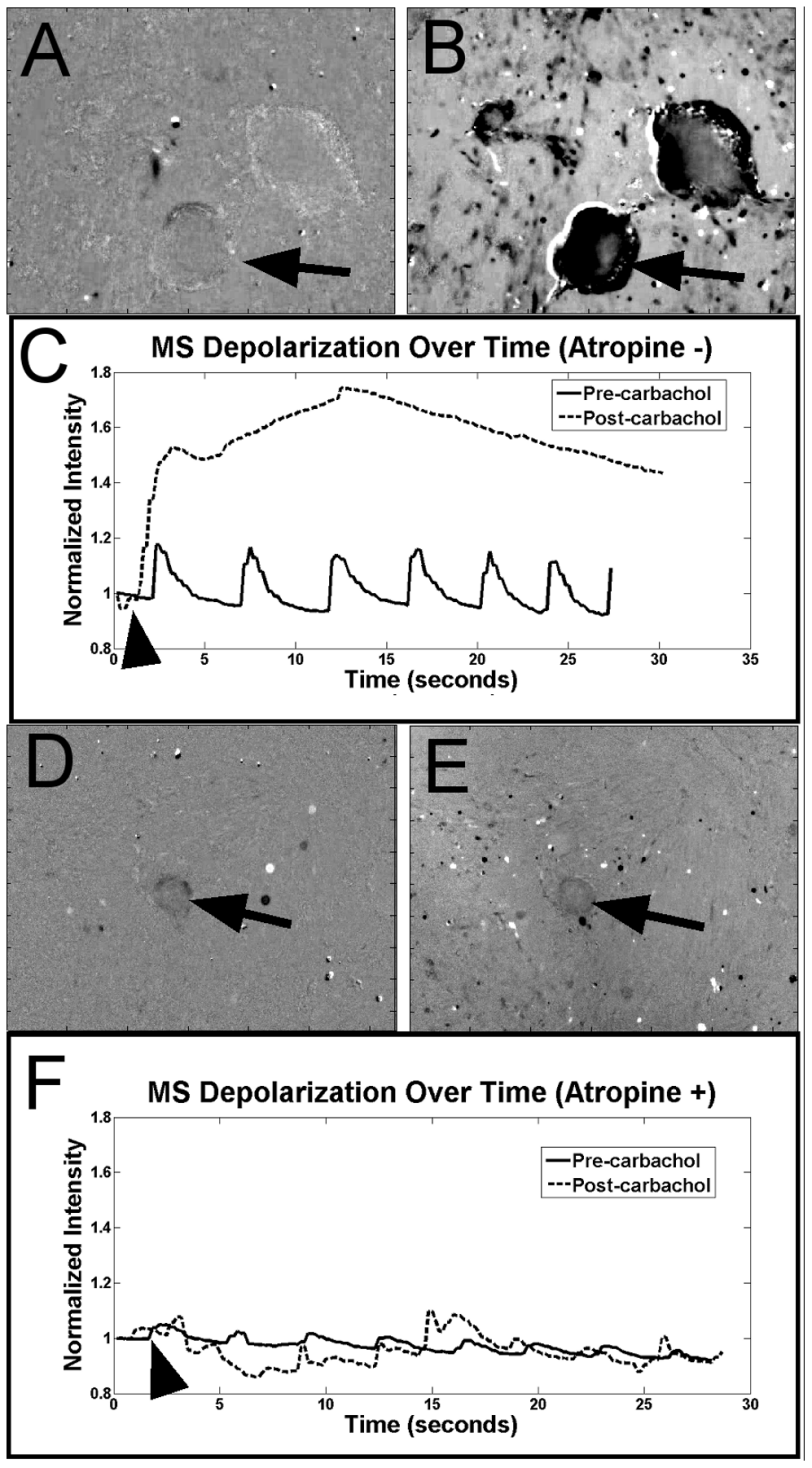
Demonstration of pharmacological response of MS to cholinergic stimulation. At day 14, the cholinergic agonist carbachol was added to cultured MS to induce MS depolarization after incubating in calcium indicator (S4 Video). Calcium intensity increased in MS between images taken just before (A) and immediately after (B) adding carbachol. A plot of calcium intensity (C) in the observed muscle strip indicates a pharmacological sensitivity to cholinergic agonists. MS were also incubated with the cholinergic antagonist atropine along with calcium indicator to reduce MS cholinergic sensitivity (S5 Video). MS with atropine were observed before (D) and after addition of carbachol (E). A plot of calcium indicator intensity shows reduced intensity after incubation in atropine compared to MS without atropine, while the addition of carbachol (arrow) did not increase intensity of the calcium indicator. An arrow points to observed MS, an arrowhead indicates addition of cholinergic stimulation on intensity profiles.100x magnification, 200-µm scale bar.

**Figure 11:**
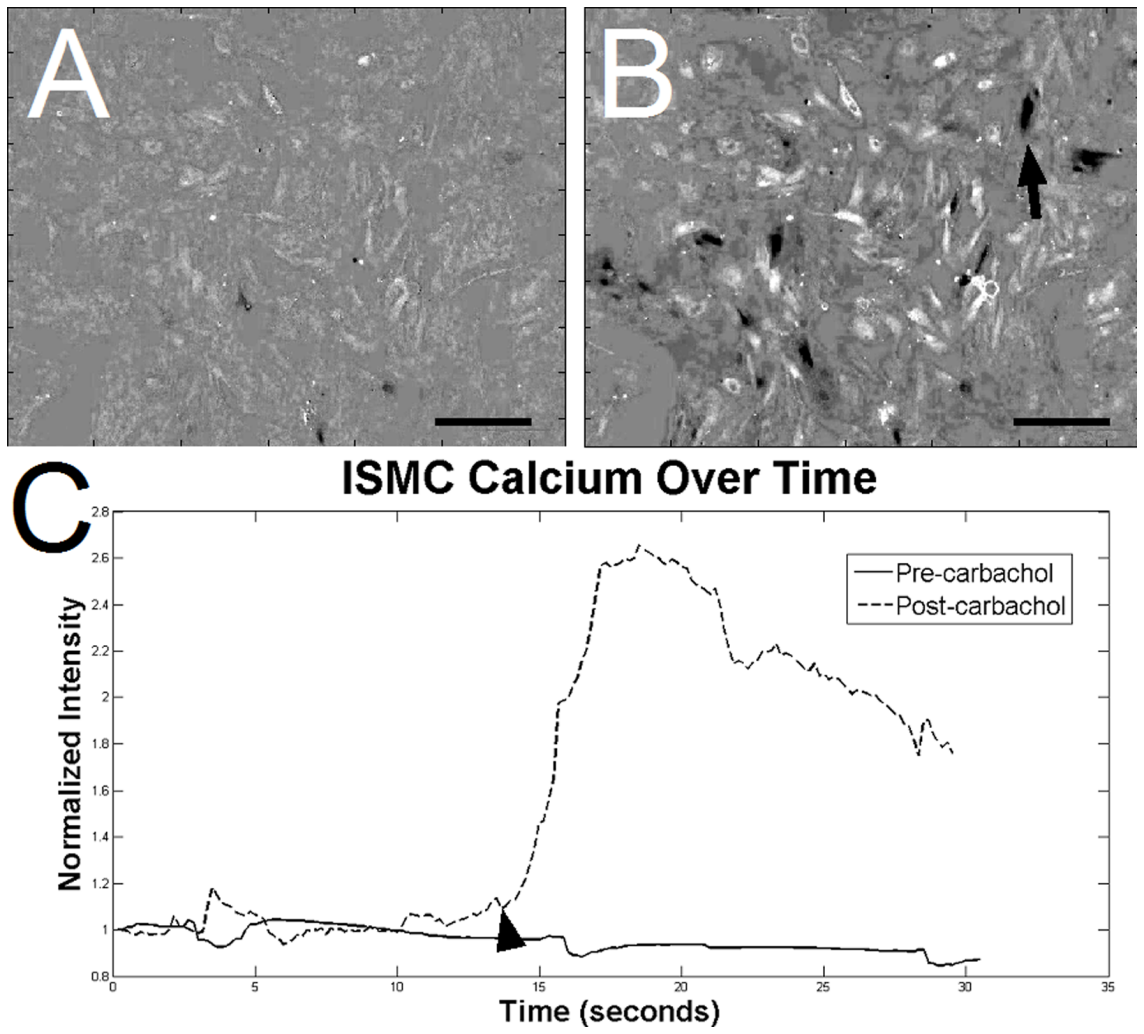
Demonstration of pharmacological response of ISMC to cholinergic stimulation. At day 14, the cholinergic agonist carbachol was added to cultured ISMC to induce depolarization after incubating in calcium indicator. Calcium intensity increased in ISMC between images taken just before (A) and immediately after (B) adding carbachol. A plot of calcium intensity (C) in the observed ISMCs indicates a pharmacological sensitivity to cholinergic agonists. An arrow points to observed MS, an arrowhead indicates addition of cholinergic stimulation on intensity profiles.100x magnification, 200-µm scale bar.

MS response to pharmacologic agents was tested with carbachol and atropine. The addition of the cholinergic agonist, carbachol, caused MS contraction and led to an increase in mean intensity within the MS and nearby clusters of cells (Fig. 10; S4 Video). Atropine, a cholinergic antagonist, did not change the periodicity of the calcium fluctuation in MS but did reduce the overall intensity (Fig. 10 D–F; S5 Video). Atropine incubation led to an attenuated response of the MS to carbachol stimulation. Cultured ISMC showed a similar response to carbachol (Fig. 11) and atropine (data not shown).

### E) Implantation of ISMC and MS

**Figure 12:**
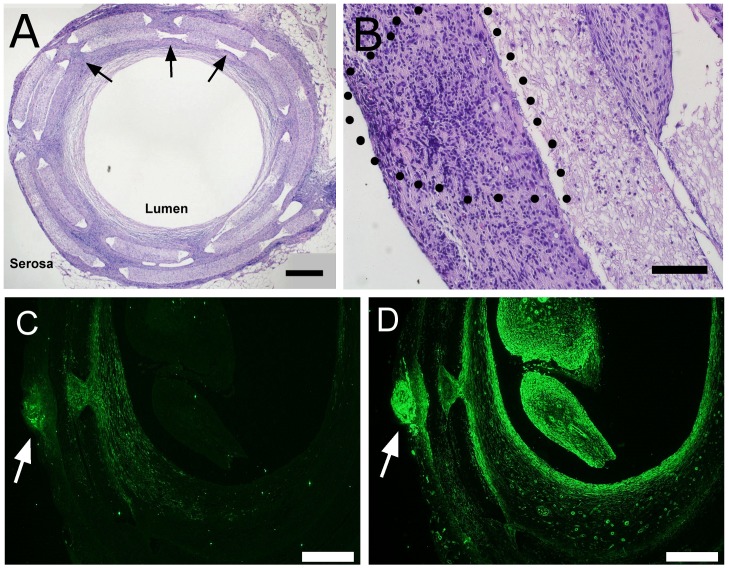
Histological analysis of implant sections. Scaffolds with either GFP-expressing ISMC or MS were retrieved after two weeks in vivo and were visualized in cross-section with H&E staining (A, B). A representative image of an ISMC-seeded explant (A, 40x, 500-µm scale) shows laser-cut pores (arrows) to improve cellular and vascular infiltration through dense PCL scaffold (PCL) from the “serosal” to luminal side of the implant. GFP-expressing MS (B, 200x, 100-µm scale) on the “serosal” surface of a PCL implant is outlined with a dotted line. Scaffolds with GFP-expressing MS were immunofluorescently labeled with antibodies to GFP or SMA (C, D). GFP-expressing and MS (C) survived the 2 week implantation and migrated out from the MS. Non-GFP-expressing cells from the host also permeated the PCL implant and had SMA immunofluorescence (D). 40x magnification, 500-µm scale bar.

**Figure 13:**
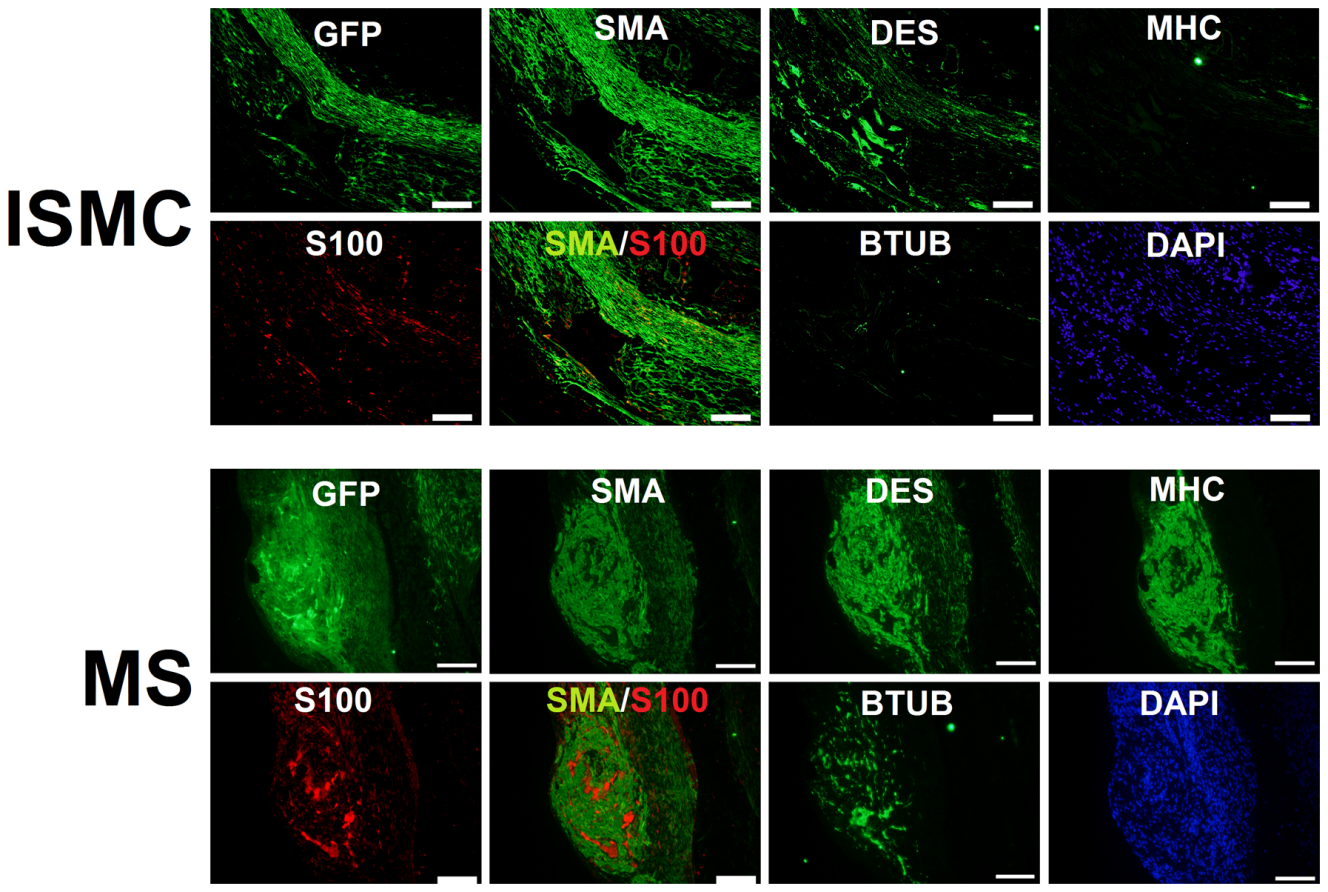
Immunofluorescent imaging of ISMC- or MS-seeded implant. After two weeks in vivo, ISMC and MS implants on scaffolds were identified with anti-GFP immunofluorescence. Both ISMC and MS expressed SMA, but ISMC had less immunofluorescence for DES and MHC compared to MS. ISMC also showed reduced S100 immunofluorescence compared to MS. Merged images of SMA (green) and s100 (red) show no co-localization of immunofluorescence in either ISMC or MS. BTUB immunofluorescence was decreased in ISMC implants compared to MS implants. Nuclei were indicated with DAPI. 200x magnification, 100-µm scale bars.

GFP-expressing ISMC and MS were seeded and cultured on macroporous, electrospun PCL scaffolds for two weeks and implanted in rat omentum to measure the persistence of mature markers *in vivo*. Rolled scaffolds were subsequently implanted in the omentum of wild-type Lewis rats for two more weeks. Retrieved implants revealed the layered structure of the scaffolds with pores and show immunofluorescence of GFP-expressing cells growing from the implanted MS (Fig. 12). Retrieved implants were also examined by immunofluorescence for smooth muscle markers, enteric nervous markers, glial markers, and GFP to identify implanted cells (Fig. 13).

GFP-expressing cells were present within the implants that were initially seeded with ISMC. These cells expressed SMA and a low level of DES (Fig. 13). Implants with MS had increased amounts of DES and MHC within the GFP-expressing MS. Implanted MS also expressed both S100 and BTUB in areas of GFP-expression, confirming a network of neural cells present within the engineered smooth muscle construct. In contrast, implanted ISMC did not express S100 or BTUB.

## Discussion

The creation of contractile smooth muscle tissues is critical to intestinal tissue engineering.

We presented a technique for forming contractile smooth muscle constructs *in vitro*, and the implanted smooth muscle construct exhibited persistent maturity and intrinsic innervation. The patterns of contraction observed in our MS are of particular interest. The period of the depolarization cycle in our MS closely resembles the period of other spontaneous depolarization cycles observed in the intestine. Mendoza et al. [19] observed a period of about 1.8 seconds per contraction for normal jejunum and up to approximately 2.5 seconds per contraction for an isolated and lengthened segment of jejunal intestine, closely resembling the 2.9 second cycles of MS cultured for 14 days. Literature values of smooth muscle periodicity report an average contraction/relaxation cycle of 1 to 3 seconds [37]. Depolarization of calcium in cultured MS followed a sweeping pattern that often migrated across the field of view or synchronized with other depolarization waves, similar to depolarization in the intestinal syncytium. Cultured ISMC had some characteristics of smooth muscle cells, but did not show the cyclical depolarization patterns observed in the MS culture.

The coordination of intestinal smooth muscle contraction is orchestrated by the enteric nervous system and the ICCs. Glial cells support the enteric nervous system through growth factor secretion and maintenance of cationic homeostasis [10], [38]. Glial cells form networks with smooth muscle cells [10], and the loss of glia decreases smooth muscle motor function in the intestine [11]. Enteric neural cells express BTUB and malformation of the enteric nervous system is one of the leading causes of gastrointestinal dysmotility [9]. Both of these cells are necessary for correct function of smooth muscle tissues in healthy intestine, and were associated with depolarization and contractile ability of the MS in this report.

Conversely, ISMC did not maintain BTUB expression measured by either mRNA expression or immunofluorescence, and ISMC were not able to contract and depolarize in culture. There was no difference in mRNA expression of either S100 or BTUB immediately following harvest (data not shown). This observation suggests that the enzymatic dissociation of MS in to ISMC either immediately killed BTUB expressing cells, or removed them from a beneficial niche within the MS that led to loss of BTUB expression and periodicity. The loss of ICC's has been associated with the loss of normal smooth muscle structure and function [39]. It is possible that pacemaking in the MS is caused by ICC's which also contribute to the maintenance of smooth muscle maturity in implanted MS [8]. Cultured MS may improve engineered smooth muscle characteristics, more research is needed to determine the cause of MS periodicity.

Many approaches to improving ISMC maturity in culture use growth factors. In a previous report, we explored the role of basic fibroblast growth factor (b-FGF) in the proliferation and survival of ISMC seeded on PCL scaffolds coated with collagen and implanted in the rat omentum [15]. Although b-FGF was able to improve cell survival within the implant, markers for maturity were not detected suggesting that ISMC still regressed to the non-contractile, “synthetic” phenotype. Therefore it is possible that maturity of ISMC may be governed by complex signaling not easily reproduced with growth factors. Similar improvements in SMC maturity have been observed in vascular SMCs co-cultured with endothelial cells [40], [41], and cultured whole strips of smooth muscle improves tissue engineered vaginal tissue [42], [43]. The lack of measurable differences in SMCs and MS suggest that differences in contractile ability in the MS may be due to tissue integrity or physical interaction between the smooth muscle cells and the pacemakers. SMCs and enteric neural cells are intimately associated and loss of this relationship during tissue dissociation may inevitably contribute to loss of SMC maturity and function (33,34).

Recently Zakhem et al. [20], [44] used a chitosan-collagen composite scaffold to engineer an aligned smooth muscle construct. The addition of pharmacological stimulants induced relevant physiological contraction or relaxation of the smooth muscle annulus. Our ISMC and MS exhibited similar physiological response to pharmacological stimulation. A follow-up study introduced enteric neural crest progenitor cells to innervate the tissue-engineered smooth muscle construct, representing a major step towards conductive engineered smooth muscle. Contractile strength of the engineered smooth muscle tissues did not replicate physiological forces of the enteric muscle. Other groups have demonstrated pharmacologically responsive smooth muscle cells with aligned morphologies but without physiological control [45]. Further progress is still needed to produce self-paced tissues capable of peristaltic motion, a significant landmark in the progress of engineered intestine. One area of recent progress utilizes thicker tissues formed from scaffolds with improved vascular infiltration, such as scaffolds with macropores [34]. Improvements toward larger, well-vascularized constructs with pacemaking ability are needed to develop engineered smooth muscle.

## Conclusion

In this study, we provide evidence that cultured smooth muscle strips maintain cellular interactions similar to those in native intestine. Our findings show that markers for enteric neural and glial cells are maintained in culture in MS as compared to dispersed ISMC. We provide evidence that MS maintains rhythmic contraction in culture, and such tissues may be useful for intestinal tissue engineering.

## Supporting Information

S1 Video **Contraction of cultured MS.** A roughly 10% change in projected area was observed on a MS cultured for 14 days, showing the retained contractile function of cultured MS. White regions indicate where MS has contracted from its original shape.
(AVI)

S2 Video **Calcium indicator intensity in cultured MS after 14 days.** A rhythmic increase in calcium indicator intensity is observed in the MS and adjacent “ganglia”. Regions of black indicate depolarization and increased intracellular calcium.
(AVI)

S3 Video **Calcium indicator intensity in cultured ISMC after 14 days.** The changes in calcium indicator of ISMC were less intense than in MS and lacked periodicity. Regions of black indicate depolarization and increased intracellular calcium.
(AVI)

S4 Video **Demonstration of pharmacological response of MS to cholinergic stimulation.** At day 14, the cholinergic agonist carbachol was added to cultured MS to induce MS depolarization after incubating in calcium indicator. Calcium intensity increased in MS during depolarization, indicating smooth muscle sensitivity to cholinergic stimulation.
(AVI)

S5 Video **Reduced sensitivity of MS to carbachol after blocking with cholinergic antagonist.** MS were also incubated with the cholinergic antagonist atropine along with calcium indicator to reduce MS cholinergic sensitivity. Carbachol was added, but MS sensitivity was reduced and cholinergic stimulation was attenuated with atropine blocking compared to carbachol stimulation alone.
(AVI)

## References

[1] Donohoe CL, Reynolds JV (2010) Short bowel syndrome. Surg J R Coll Surg Edinburgh Irel 8:270–279. 10.1016/j.surge.2010.06.004 20709285

[2] Bines JE (2009) Intestinal failure: A new era in clinical management. J Gastroenterol Hepatol 24 Suppl 3S86–92. 19799705 10.1111/j.1440-1746.2009.06077.x

[3] Weih S, Kessler M, Fonouni H, Golriz M, Hafezi M, et al (2011) Current practice and future perspectives in the treatment of short bowel syndrome in children-a systematic review. Langenbecks Arch Surg. 10.1007/s00423-011-0874-8 22105773

[4] Tortora GJ, Derrickson BH (2008) Principles of Anatomy and Physiology. 12th Edition. Wiley.

[5] Marieb EN, Hoehn K (2004) Human Anatomy & Physiology. 6th Edition. New York: Pearson Education.

[6] Goyal RK, Chaudhury A (2010) Mounting evidence against the role of ICC in neurotransmission to smooth muscle in the gut. Am J Physiol Gastrointest Liver Physiol 298:G10–G13. 19892937 10.1152/ajpgi.00426.2009 PMC2806097

[7] Sanders KM (1996) A Case for Interstitial Cells of Cajal as Pacemakers and Mediators of Neurotransmission in the Gastrointestinal Tract. Gastroenterology 111:492–515. 8690216 10.1053/gast.1996.v111.pm8690216

[8] Sarna SK (2008) Are interstitial cells of Cajal plurifunction cells in the gut? Am J Physiol Gastrointest Liver Physiol 294:G372–390. 17932226 10.1152/ajpgi.00344.2007

[9] Gabella G (2012) Cells of visceral smooth muscles. J Smooth Muscle Res 48:65–95. 23095736 10.1540/jsmr.48.65

[10] Rühl A (2005) Glial cells in the gut. Neurogastroenterol Motil 17:777–790. 16336493 10.1111/j.1365-2982.2005.00687.x

[11] Nasser Y, Fernandez E, Keenan CM, Ho W, Oland LD, et al (2006) Role of enteric glia in intestinal physiology: effects of the gliotoxin fluorocitrate on motor and secretory function. Am J Physiol Gastrointest Liver Physiol 1:912–927. 10.1152/ajpgi.00067.2006 16798727

[12] Thyberg J (1996) Differentiated properties and proliferation of arterial smooth muscle cells in culture. Int Rev Cytol: 183–265. 8843655 10.1016/s0074-7696(08)61987-7

[13] Hellstrand P, Albinsson S (2005) Stretch-dependent growth and differentiation in vascular smooth muscle: role of the actin. 875:869–875. 10.1139/y05-061 16333359

[14] Nair DG, Han TY, Lourenssen S, Blennerhassett MG (2011) Proliferation modulates intestinal smooth muscle phenotype in vitro and in colitis in vivo. Am J Physiol Gastrointest Liver Physiol 300:G903–13. 21311027 10.1152/ajpgi.00528.2010

[15] Lee M, Wu BM, Stelzner M, Reichardt HM, Dunn JCY (2008) Intestinal smooth muscle cell maintenance by basic fibroblast growth factor. Tissue Eng Part A 14:1395–1402. 18680389 10.1089/ten.tea.2007.0232

[16] Sobue K, Hayashi K, Nishida W (1999) Expressional regulation of smooth muscle cell-specific genes in association with phenotypic modulation. Mol Cell Biochem 190:105–118. 10098977

[17] Shi X-Z, Sarna SK (2013) Cell culture retains contractile phenotype but epigenetically modulates cell-signaling proteins of excitation-contraction coupling in colon smooth muscle cells. Am J Physiol Gastrointest Liver Physiol 304:G337–45. 23238936 10.1152/ajpgi.00369.2012 PMC3566616

[18] Owens GK (1995) Regulation of differentiation of vascular smooth muscle cells. Physiol Rev 75:487–517. 7624392 10.1152/physrev.1995.75.3.487

[19] Mendoza J, Chang C, Blalock CL, Atkinson JB, Wu BM, et al (2006) Contractile function of the mechanically lengthened intestine. J Surg Res 136:8–12. 16979663 10.1016/j.jss.2006.01.027

[20] Zakhem E, Raghavan S, Gilmont RR, Bitar KN (2012) Chitosan-based scaffolds for the support of smooth muscle constructs in intestinal tissue engineering. Biomaterials 33:4810–4817. 22483012 10.1016/j.biomaterials.2012.03.051 PMC3334429

[21] Moon DG, Christ G, Stitzel JD, Atala A, Yoo JJ (2008) Cyclic mechanical preconditioning improves engineered muscle contraction. Tissue Eng Part A 14:473–482. 18399787 10.1089/tea.2007.0104

[22] Ott HC, Matthiesen TS, Goh S-K, Black LD, Kren SM, et al (2008) Perfusion-decellularized matrix: using nature's platform to engineer a bioartificial heart. Nat Med 14:213–221. 18193059 10.1038/nm1684

[23] Yazdani SK, Watts B, Machingal M, Jarajapu YPR, Van Dyke ME, et al (2009) Smooth muscle cell seeding of decellularized scaffolds: the importance of bioreactor preconditioning to development of a more native architecture for tissue-engineered blood vessels. Tissue Eng Part A 15:827–840. 19290806 10.1089/ten.tea.2008.0092

[24] Miyasaka EA, Raghavan S, Gilmont RR, Mittal K, Somara S, et al (2011) In vivo growth of a bioengineered internal anal sphincter: comparison of growth factors for optimization of growth and survival. Pediatr Surg Int 27:137–143. 21046117 10.1007/s00383-010-2786-z PMC3022992

[25] Jack GS, Zhang R, Lee M, Xu Y, Wu BM, et al (2009) Urinary bladder smooth muscle engineered from adipose stem cells and a three dimensional synthetic composite. Biomaterials 30:3259–3270. 19345408 10.1016/j.biomaterials.2009.02.035 PMC2744495

[26] Stanzel RDP, Lourenssen S, Nair DG, Blennerhassett MG (2010) Mitogenic factors promoting intestinal smooth muscle cell proliferation. Am J Physiol Cell Physiol 299:C805–17. 20631246 10.1152/ajpcell.00086.2010

[27] Nakase Y, Hagiwara A, Nakamura T, Kin S, Nakashima S, et al (2006) Tissue engineering of small intestinal tissue using collagen sponge scaffolds seeded with smooth muscle cells. Tissue Eng 12:403–412. 16548698 10.1089/ten.2006.12.403

[28] Lee M, Wu BM, Dunn JCY (2008) Effect of scaffold architecture and pore size on smooth muscle cell growth. J Biomed Mater Res A 87:1010–1016. 18257081 10.1002/jbm.a.31816

[29] Sala FG, Matthews JA, Speer AA, Torashima Y, Barthel ER, et al (2011) A Multicellular Approach Forms a Significant Amount of Tissue-Engineered Small Intestine in the Mouse. Tissue Eng Part A 17:1841–1850. 21395443 10.1089/ten.tea.2010.0564 PMC3118603

[30] Alexakis C, Guettoufi A, Mestries P, Strup C, Mathé D, et al (2001) Heparan mimetic regulates collagen expression and TGF-beta1 distribution in gamma-irradiated human intestinal smooth muscle cells. FASEB J 15:1546–1554. 11427486 10.1096/fj.00-0756com

[31] Cha JM, Park S, Park G, Kim JK, Suh H (2006) Construction of functional soft tissues from premodulated smooth muscle cells using a bioreactor system. Artif Organs 30:704–707. 16934099 10.1111/j.1525-1594.2006.00287.x

[32] Gutierrez JA, Perr HA (1999) Mechanical stretch modulates TGF-β1 and αlpha1(I) collagen expression in fetal human intestinal smooth muscle cells. Am J Physiol Gastrointest Liver Physiol 277:G1074–1080. 10.1152/ajpgi.1999.277.5.G1074 10564114

[33] Walthers CM, Nazemi AK, Patel SL, Wu BM, Dunn JCY (2014) The effect of scaffold macroporosity on angiogenesis and cell survival in tissue-engineered smooth muscle. Biomaterials 35:5129–5137 doi:10.1016/j.biomaterials.2014.03.025 24695092 10.1016/j.biomaterials.2014.03.025 PMC4018739

[34] Joshi VS, Lei NY, Walthers CM, Wu B, Dunn JCY (2013) Macroporosity enhances vascularization of electrospun scaffolds. J Surg Res 183:18–26. 23769018 10.1016/j.jss.2013.01.005 PMC3694756

[35] Geisbauer CL, Chapin JC, Wu BM, Dunn JCY (2012) Transplantation of enteric cells expressing p75 in the rodent stomach. J Surg Res 174:257–265. 21324400 10.1016/j.jss.2010.12.016

[36] Altomare A, Gizzi A, Guarino MPL, Loppini A, Cocca S, et al (2014) Experimental evidence and mathematical modeling of thermal effects on human colonic smooth muscle contractility. Am J Physiol Gastrointest Liver Physiol 307:G77–88. 24833706 10.1152/ajpgi.00385.2013

[37] Guyton AC, Hall JE (2000) Membrane Physiology, Nerve, and Muscle. In: Schmitt W, Gruliow R, editors. Textbook of Medical Physiology. Philadelphia, PA: W.B. Saunders Company. pp.87–94.

[38] Gabella G (1981) Ultrastructure of the nerve plexuses of the mammalian intestine: the enteric glial cells. Neuroscience 6:425–436. 7219723 10.1016/0306-4522(81)90135-4

[39] Tennyson VM, Pham TD, Rothman TP, Gershon MD (1986) Abnormalities of smooth muscle, basal laminae, and nerves in the aganglionic segments of the bowel of lethal spotted mutant mice. Anat Rec 215:267–281. 3740466 10.1002/ar.1092150310

[40] Neff LP, Tillman BW, Yazdani SK, Machingal MA, Yoo JJ, et al (2011) Vascular smooth muscle enhances functionality of tissue-engineered blood vessels in vivo. J Vasc Surg 53:426–434. 20934837 10.1016/j.jvs.2010.07.054

[41] Bulick AS, Muñoz-Pinto DJ, Qu X, Mani M, Cristancho D, et al (2009) Impact of endothelial cells and mechanical conditioning on smooth muscle cell extracellular matrix production and differentiation. Tissue Eng Part A 15:815–825. 19108675 10.1089/ten.tea.2008.0179

[42] De Filippo RE, Yoo JJ, Atala A (2003) Engineering of vaginal tissue in vivo. Tissue Eng 9:301–306. 12740092 10.1089/107632703764664765

[43] De Filippo RE, Bishop CE, Filho LF, Yoo JJ, Atala A (2008) Tissue engineering a complete vaginal replacement from a small biopsy of autologous tissue. Transplantation 86:208–214. 18645481 10.1097/TP.0b013e31817f1686

[44] Zakhem E, Raghavan S, Bitar KN (2014) Neo-innervation of a bioengineered intestinal smooth muscle construct around chitosan scaffold. Biomaterials 35:1882–1889. 24315576 10.1016/j.biomaterials.2013.11.049

[45] Somara S, Gilmont RR, Dennis RG, Bitar KN (2009) Bioengineered internal anal sphincter derived from isolated human internal anal sphincter smooth muscle cells. Gastroenterology 137:53–61. 19328796 10.1053/j.gastro.2009.03.036

